# ‘Aerobic’ and ‘Anaerobic’ terms used in exercise physiology: a critical terminology reflection

**DOI:** 10.1186/s40798-015-0012-1

**Published:** 2015-03-27

**Authors:** Karim Chamari, Johnny Padulo

**Affiliations:** 1 Athlete Health and Performance Research Centre, ASPETAR, Qatar Orthopaedic and Sports Medicine Hospital, Doha, Qatar; 2 University e-Campus, Via Isimbardi, 10-22060 Novedrate, CO Italy; 3 Tunisian Research Laboratory “Sports Performance Optimization”, National Center of Medicine and Science in Sports, Tunis, Tunisia

## Abstract

The purpose of this Current Opinion article is to focus on the appropriate use of the terms ‘aerobic’- and ‘anaerobic’-exercise in sports medicine, in order to try to unify their use across coaches/athletes and sport scientists. Despite the high quality of most of the investigations, the terms aerobic/anaerobic continue to be used inappropriately by some researchers in exercise science. Until late 2014, for instance, 14,883 and 6,136 articles were cited in PubMed, in the field of ‘exercise science’, using the words ‘aerobic’ or ‘anaerobic’, respectively. In this regard, some authors still misuse these terms. For example, we believe it is wrong to classify an effort as ‘anaerobic lactic exercise’ when other metabolic pathways are also simultaneously involved. It has extensively been shown that the contribution of the metabolic pathways mainly depends on both exercise intensity and duration. Therefore, it is our intent to further clarify this crucial point and to simplify this terminology for coaches and sports scientists. In this regard, several research articles are discussed in relation to the terminology used to describe the predominant metabolic pathways active at different exercise durations and the oversimplification this introduces. In conclusion, we suggest that sports scientists and field practitioners should use the following terms for *all-out* (‘*maximal*’*)* efforts based on *exercise duration*: (a) ‘Explosive Efforts’ (duration up to 6 s, with preponderance of the ‘phosphagens’ metabolic pathway’); (b) ‘High Intensity Efforts’ (efforts comprised between >6 s and 1 min, with preponderance of the ‘glycolytic pathway’), and (c) ‘Endurance Intensive Efforts’ (for exercise bouts longer than 1 min, with preponderance of the ‘oxidative phosphorylation pathway’).

## Key Points

- Appropriate use of terms ‘aerobic’ and ‘anaerobic’ in Exercise Science is discussed.
- Metabolic contributions to exercise cannot be so easily separated or categorized; therefore, it is advisable to remove them when naming physical efforts.
- The *All-out* (‘maximal’) efforts could be categorized in ‘Explosive’, ‘High-Intensity’, or ‘Endurance-Intensive’ - Efforts based on exercise duration.

## Background

Sport science and sport practice on the field are tightly linked. Indeed, researchers are often inspired by sport performance and training facts, while sport practitioners (athletes, coaches, physicians, physiotherapists…) extensively use sport science in their daily practice. Ideally, both sides should use common terms to avoid any misunderstanding that could be translated to, among other things, inappropriate training. In that regard, in 1978, Knuttgen [1] published a pioneering study which proposed the term ‘intensity’ to describe the hardness with which the exercise is performed or perceived as percentage of the external load. Some 30 years later, a book by Winter and MacLaren [2] highlighted this paradigm using ‘*maximal-intensity exercise*’ to describe the exercise physiology related to aerobic/anaerobic metabolism’s contribution to energy supply. In this context, we believe that describing efforts/exercise based on their ‘physiological pathway’ could lead to mistakes. Indeed, many authors describe short-duration exercise as ‘*anaerobic*’ [3-6] and longer efforts as ‘*aerobic*’ [3-7]. Nevertheless, exercise physiology has evolved during the last decade, and technology has contributed to evolve from Douglas bags in the laboratory to portable gas analyzers in the field to assess cardiorespiratory responses to exercise [8]. Moreover, invasive methods such as muscle biopsies allow for researchers to clarify the kinetics of the aerobic/anaerobic metabolism during exercise [9,10]. Henceforth, portable and affordable blood lactate analyzers [11] allow the researchers to easily measure lactate (a metabolite of glycolysis) and to assess the extent of ‘anaerobic’ contribution to exercise, in the field within seconds [12]. Despite that, some recent publications still report nomenclatures that are not up to date [13].

**Figure 1:**
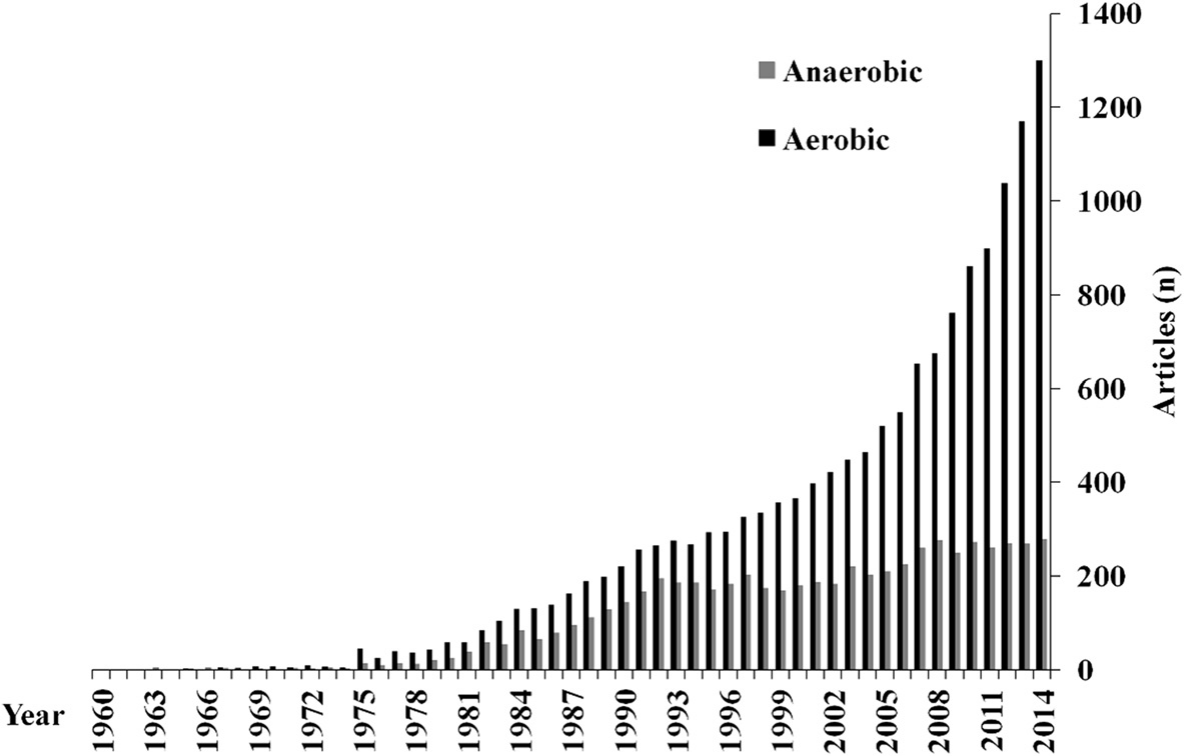
**Use of terms ‘** ***aerobic*** **and** ***anaerobic*** **’ in exercise science on Pubmed as of December 2014.**

The purpose of the present ‘Current Opinion article’ is therefore to highlight the errors underlying particular nomenclature. This should help to standardize the terminologies published by scientists across the world. In that regard, the aerobic/anaerobic terminology in sport science (Figure 1) raises some issues for the following reasons:

- The term ‘anaerobic’ is misunderstood - some think it refers to the absence of O_2_. The term ‘aerobic’ also seems implying an absence of any ‘anaerobic’ contribution.
- Metabolic contributions to exercise cannot be so easily separated or categorized.
- The intensity of the exercise highly impacts upon the metabolic contribution of the energy pathways; therefore, a clarification should be done in that regard.
- Some sport practitioners could have a limited ‘physiology background’ and, therefore, increases the likelihood of misusing terms and concepts in their field practice.

In recent times, a number of key publications have challenged former concepts resulting in a change in opinions and understandings. For example, Racz et al. (2002) [14] investigated muscle contraction and brought new data to the view-point of muscle contraction as represented by Hill in 1938 [15]. In the same context, a number of recent articles highlighted in this ‘Current Opinion Article’ have allowed us to ‘consider’ efforts in a different way than the way they were first described some decades ago.

It is important to note that the ‘anaerobic’ metabolism is not a pathway that functions in the absence of oxygen but rather it ‘does not use oxygen’. The ‘anaerobic’ metabolism that transforms adenosine triphosphate (ATP) and phosphocreatine (CrP) should therefore not be termed ‘anaerobic’ but rather ‘independent of oxygen’ or ‘non-mitochondrial’ [16]. Thus, instead of calling it the ‘anaerobic a-lactic pathway’, it should be termed the ‘phospagens’ pathway’. Likewise, ‘glycolysis’ should simply replace the ‘anaerobic lactic pathway’, as again although not directly involved in this pathway, oxygen is still present. For the third metabolic energy pathway, ‘oxidative phosphorylation’ should replace the term ‘aerobic pathway’. Furthermore, at the laboratory, attempts to quantify contributions from anaerobic/aerobic metabolisms are bedevilled by technical and theoretical challenges that have been addressed quite successively by several research groups [7,10,17]. For instance, Medbo et al. (1988) have presented a method to assess anaerobic metabolism capacity [17]. This method, even though being based on sound theory, has been further criticized for the fact that over VO_2max_, the power/VO_2_ relationship is no longer linear [17,18].

## Discussion

Very short all-out efforts (lasting less than 1 s to around 6 s) are not only dependent on the phosphagen pathway, but also partially on glycolysis [19,20]. For example, one single ‘maximal’ 6-s sprint is in fact performed with approximately half the energy originating from ‘phosphagens’ while the other half is originating from ‘glycolytic’ pathways [20]. This finding of Gaitanos et al. [20] was published more than 20 years ago, and we believe it is time to take it into account when understanding short ‘all-out’ efforts. The latter efforts are exercise bouts during which the athlete tries to reach the highest performance possible for the pre-determined effort duration [21]. Therefore, instead of calling these efforts as ‘anaerobic a-lactic exercises’, they should be called, for example, ‘short-term high intensity efforts’ or, in a shorter way, ‘explosive efforts’. These explosive efforts are performed at power outputs approximately sixfold higher than that of ‘maximal aerobic power (MAP; which is discussed in further detail below)’ [2]. Moreover, years ago, longer all-out efforts of less than 1-min duration were described as ‘anaerobic’; a claim based on (a) a theoretical equation [22] and (b) on the oxygen uptake measured during the first minute of exercise [23]. However, Spencer et al. [21], amongst others, demonstrated mixed anaerobic/aerobic contributions in different exercise durations (from 20 to 234 s) corresponding to racing distances ranging from 200 to 1,500 m. Several authors [6,7] showed that even in very short all-out field and laboratory efforts a significant contribution from ‘oxidative phosphorylation’ (which is also called ‘aerobic metabolism’) was also present [16]. In particular, this relative contribution increases further when sprints are repeated [24].

On the field, endurance efforts are often described as ‘aerobic’. However, purely aerobic exercise does not exist as long as a minimum of intensity is put into the efforts. In this context, it is incorrect to call the considered ‘gold-standard’ test used for assessing aerobic capability/fitness, i.e., ‘the maximal oxygen uptake (VO_2max_) test’, an ‘aerobic test’. In this regard, recent studies challenge the concept of VO_2max_ after modifications to the test protocol allowed attainment of different VO_2max_ values [25]. Indeed, one of the criteria for the attainment of the VO_2max_ plateau is to reach a minimum value for Lactate of 6 to 9 mmol L^−1^ (depending on the authors and the age of the subjects). This clearly shows a significant participation of ‘Glycolysis’ prior to the cessation of exercise. This is not surprising, as a maximal effort at the end of a ‘VO_2max_ test’ occurs at intensities well beyond the second ventilatory threshold (which is also described as respiratory compensation threshold [26]). Therefore, we believe that every exercise should be described for what it is specifically assessing thereby avoiding erroneously describing particular metabolic pathway(s) involved. For instance, to describe an incremental test (VO_2max_) outcome, one cannot speak of the ‘maximal aerobic speed’ reached, but of the ‘peak speed reached at VO_2max_’ or ‘*vpeak*VO_2max_’ as justly used by Billat et al. [27].

Moreover, there have been lacks of quantification of the contribution of the anaerobic energy [2] to discriminate percentage of anaerobic versus aerobic metabolism during an effort. To clarify this gap, 40 years ago, Hermansen proposed for the first time an indirect estimation of anaerobic capacity by the ‘*maximal accumulated oxygen deficit* (MAOD) assessment’ based on maximal intensity exercise and gas exchange measures [28]. Several years later, the MAOD method was further experimented by Mebdo et al. [17], even though this method also raises some small methodological issues (mentioned above), it is now possible to estimate anaerobic and aerobic contributions to exercise. In that regard, it has been too often suggested that ‘aerobic’ metabolism contributes to the provision of exercise energy several seconds/minutes after the start of exercise. However, Granier et al. (1995) showed that for a 30-s *all-out* exercise (Wingate-test, firstly presented as a way of assessing anaerobic capacity [29]), the contribution of this pathway varies from 28% to 45% of total energy production (depending on the profile of the athletes [7]), showing again a misnomer in exercise physiology/testing [2]. Furthermore, during a 400-m *all-out* run of about 52-s, the last 20-s of effort is performed at VO_2max_, showing that the activation of ‘oxidative phosphorylation’ is much faster than previously thought [21]. Today, it is accepted that the energy provision for every effort relies on the simultaneous participation of all three energy pathways with a predominant pathway working above the others [21]. Therefore, describing the efforts should not be based on their ‘physiological processes’, but rather they should be called in accordance to their duration/intensity. More specifically, for ‘all-out efforts’ (maximal effort for the pre-determined duration), we propose to call

1. ‘Explosive Efforts’: all-out exercises with a duration of up to 6 s (predominance of ‘phosphagens’ pathway’).
2. ‘High Intensity Efforts’: all-out efforts lasting from 6 s to 1 min [21] (predominance of the ‘glycolytic pathway’ in addition to the ‘phosphagen’s pathway’ and ‘oxidative phosphorylation’); and finally,
3. ‘Endurance Intensive Efforts’: exercise with a duration exceeding 1 min (predominance of ‘oxidative phosphorylation’).

For sub-maximal intensity exercise, other definitions also need to be proposed. In that regard, the paradigm of aerobic and anaerobic metabolism is in need of further research, with both systems complementing each other. In fact, ‘aerobic’ is often intended as ‘uses oxygen’, whereas ‘anaerobic’ as ‘does not use oxygen’. That’s why any misuse of the terms may lead to misleading concepts and misunderstanding for the readers, and potential mistakes on the field for training prescription. We believe that some other concepts of exercise physiology in *sport science* still need similar clarification, and we encourage expert colleagues to clarify these points in relevant consensus statements. This would help sport and exercise science evolve in the right direction, using appropriate terminology that helps scientists, coaches, teachers, and students to speak the same language [30].

## Conclusions

In summary, instead of calling an exercise effort anything related to ‘aerobic’ and/or ‘anaerobic’ physiological pathways, even if a further research studies might be needed to improve our proposal, we suggest that sports scientists should use the following terms for *all-out* (maximal) efforts based on *exercise duration*:

1. ‘Explosive Efforts’ (duration up to 6 s)
2. ‘High Intensity Efforts’ (efforts comprised between >6 s to 1 min)
3. ‘Endurance Intensive Efforts’ (for exercise bouts longer than 1 min)

## References

[1] Knuttgen HG (1978). Force, work, power, and exercise. Med Sci Sports.

[2] Winter EM, MacLaren DP, Eston R, Reilly T (2009). Assessment of maximal-intensity exercise. Kinanthropometry and Exercise Physiology Laboratory Manual Volume 2: Physiology (third edition).

[3] Chaouachi A, Coutts AJ, Chamari K, Wong DP, Chaouachi M, Chtara M (2009). Effect of Ramadan intermittent fasting on aerobic and anaerobic performance and perception of fatigue in male elite judo athletes. J Strength Cond Res.

[4] Chtourou H, Hammouda O, Chaouachi A, Chamari K, Souissi N (2012). The effect of time-of-day and Ramadan fasting on anaerobic performances. Int J Sports Med.

[5] Rodriguez-Marroyo JA, Garcia-Lopez J, Chamari K, Cordova A, Hue O, Villa JG (2009). The rotor pedaling system improves anaerobic but not aerobic cycling performance in professional cyclists. Eur J Appl Physiol.

[6] Billat V, Renoux JC, Pinoteau J, Petit B, Koralsztein JP (1995). Times to exhaustion at 90, 100 and 105% of velocity at VO2 max (maximal aerobic speed) and critical speed in elite long-distance runners. Arch Physiol Biochem.

[7] Granier P, Mercier B, Mercier J, Anselme F, Prefaut C (1995). Aerobic and anaerobic contribution to Wingate test performance in sprint and middle-distance runners. Eur J Appl Physiol Occup Physiol.

[8] McLaughlin JE, King GA, Howley ET, Bassett DR, Ainsworth BE (2001). Validation of the COSMED K4 b2 portable metabolic system. Int J Sports Med.

[9] Bangsbo J, Gollnick PD, Graham TE, Juel C, Kiens B, Mizuno M (1990). Anaerobic energy production and O2 deficit-debt relationship during exhaustive exercise in humans. J Physiol.

[10] Bangsbo J (1996). Oxygen deficit: a measure of the anaerobic energy production during intense exercise?. Can J Appl Physiol.

[11] Baldari C, Bonavolonta V, Emerenziani GP, Gallotta MC, Silva AJ, Guidetti L (2009). Accuracy, reliability, linearity of Accutrend and Lactate Pro versus EBIO plus analyzer. Eur J Appl Physiol.

[12] Hertogh C, Chamari K, Damiani M, Martin R, Hachana Y, Blonc S (2005). Effects of adding a preceding run-up on performance, blood lactate concentration and heart rate during maximal intermittent vertical jumping. J Sports Sci.

[13] Wells GD, Selvadurai H, Tein I (2009). Bioenergetic provision of energy for muscular activity. Paediatr Respir Rev.

[14] Racz L, Beres S, Hortobagyi T, Tihanyi J (2002). Contraction history affects the in vivo quadriceps torque-velocity relationship in humans. Eur J Appl Physiol.

[15] Hill AV (1938). The heat of shortening and the dynamic constants of muscle. Proceedings of the Royal Society of London Series B, Biological Sciences.

[16] Baker JS, McCormick MC, Robergs RA (2010). Interaction among skeletal muscle metabolic energy systems during intense exercise. J Nutr Metab.

[17] Medbo JI, Mohn AC, Tabata I, Bahr R, Vaage O, Sejersted OM (1988). Anaerobic capacity determined by maximal accumulated O2 deficit. J Appl Physiol (1985).

[18] Chamari K, Chaouachi A, Racinais S, P Connes OHaSP (2010). Anaerobic power and capacity. Exercise Physiology: from a Cellular to an Integrative Approach.

[19] Chamari K, Ahmaidi S, Blum JY, Hue O, Temfemo A, Hertogh C (2001). Venous blood lactate increase after vertical jumping in volleyball athletes. Eur J Appl Physiol.

[20] Gaitanos GC, Williams C, Boobis LH, Brooks S (1993). Human muscle metabolism during intermittent maximal exercise. J Appl Physiol (1985).

[21] Spencer MR, Gastin PB (2001). Energy system contribution during 200- to 1500-m running in highly trained athletes. Med Sci Sports Exerc.

[22] Hartree W, Hill AV (1923). The anaerobic processes involved in muscular activity. J Physiol.

[23] Astrand PO, Saltin B (1961). Oxygen uptake during the first minutes of heavy muscular exercise. J Appl Physiol.

[24] Glaister M (2005). Multiple sprint work: physiological responses, mechanisms of fatigue and the influence of aerobic fitness. Sports Med.

[25] Beltrami FG, Froyd C, Mauger AR, Metcalfe AJ, Marino F, Noakes TD (2012). Conventional testing methods produce submaximal values of maximum oxygen consumption. Br J Sports Med.

[26] Beaver WL, Wasserman K, Whipp BJ (1986). A new method for detecting anaerobic threshold by gas exchange. J Appl Physiol (1985).

[27] Billat VL, Blondel N, Berthoin S (1999). Determination of the velocity associated with the longest time to exhaustion at maximal oxygen uptake. Eur J Appl Physiol Occup Physiol.

[28] Hermansen L (1969). Anaerobic energy release. Med Sci Sports Exerc.

[29] Bar-Or O (1987). The Wingate anaerobic test. An update on methodology, reliability and validity. Sports Med.

[30] Winter EM, Fowler N (2009). Exercise defined and quantified according to the Systeme International d'Unites. J Sports Sci.

